# Supplementary material for: Multidisciplinary Team Care in Pituitary Tumours
Source: Cancers (Basel). 2024 Feb 27;16(5):950. doi: 10.3390/cancers16050950 (PMC10930925; doi:10.3390/cancers16050950)
Supplement: Supplementary file 1 [file cancers-16-00950-s001.zip › cancers-2747657-supplementary.pdf]

Supplementary Table S1: The perspectives of different specialists involved in our Pituitary MDT regarding the question "What is the major contribution that your specialty can provide to the Pituitary Multidisciplinary Team?"

Medical specialist	What is the major contribution that your specialty can provide to the Pituitary Multidisciplinary Team (MDT)?
**Neurosurgeon**	"Nowadays neurosurgery, as many other surgical disciplines, has achieved a high complexity standard of care due to significant improvement in the diagnostic tools and technological applied devices in the operating room, as well as due to increased knowledge about aspects of the nervous system and its related diseases. It has been necessary to set-up specialisation programs to offer the best treatment to each neurosurgical case. We need to work as a team in the decision-making workflow for a real patient-centered care. The creation of MDT in pituitary diseases is an example of the need to meet all the disciplines involved from the diagnosis to the follow-up after surgical treatment to achieve the best outcomes for the patient. The complex needs of patients, families and communities are not usually amenable to the actions of a single care provider in isolation from colleagues of other disciplines. Knowledge exchange and teamwork focused to each patient achieve better results, less complications and a better use of resources. The major contribution of a neurosurgeon is delivering the best surgical outcomes with the smaller number of complications, which will facilitate the task of other colleagues, such as the endocrinologists."
**Endocrinologist**	"In the realm of pituitary MDT care, endocrinology stands as a linchpin providing invaluable insights and expertise. Since many pituitary tumours affect hormone-producing cells within the pituitary, endocrinologists are trained and thus possess a unique skill set for assessing and diagnosing hormonal imbalances, including hormone excess or deficiency arising from the tumour or its treatment. Endocrinologists are experts in managing therapies to restore hormonal balance, providing personalised hormone replacements, supporting patients during peri-operative care and long-term follow-up. Based on hormone levels, tumour size, location, and patient-specific factors, endocrinologists must work closely with other members to develop the most suitable treatment approach, which can be surgery, radiation, medical therapy, or a combination of all of these. Pituitary tumours often require long-term care and surveillance for recurrence. Endocrinologists coordinate follow-up care, ensuring that patients undergo regular tests and evaluations. This unwavering vigilance optimizes outcomes and minimizes adverse effects. Endocrinologists also play a key role in the education of patients and their families about the condition, treatment options and lifestyle modifications, which is a crucial support to help patients managing their condition in the long-term and improving their quality of life."
**Neuroradiologist**	"Neuroradiologists play a critical role in a Pituitary MDT and should possess specific knowledge and skills to contribute to the team's success. The contribution goes beyond reading scans and can be felt in other steps of the process. Neuroradiologists should have a deep knowledge on pituitary anatomy, including its relationship with adjacent structures, and experience on choosing/advising the team for the most adequate examination in specific clinical scenarios. Neuroradiologists must facilitate the easy access to quality and up-to-date imaging techniques, such as high field MRI, with high

	resolution images and capability for dynamic sequences, multi-slice high-resolution CT scans and a Digital Subtraction Angiography for collecting direct microcatheter pituitary blood samples. During the diagnosis it is important to localise the lesions, to characterise their size, shape, content, and relationship with adjacent structures. Neuroradiologists should be able to discuss differential diagnosis for sellar and suprasellar masses. The communication with the team should be clear and concise, the scans must be presented and explained during the MDT meetings and the reports should follow a standardized model. For patients requiring surgery, neuroradiologists may help the surgeon in planning safe approaches and in providing CT and MR3D neuronavigation images. Neuroradiologists may also have an important contribution for treatment monitoring. After surgery or other treatments, serial imaging helps assessing treatment response, recurrence or development of complications."
**Neuropathologist**	"The approach to the pathology of the pituitary and sellar region is complex because this area may be affected by many tumours and pseudotumoural lesions, so knowledge of multiple pathological conditions is required. The first decision to be taken with a surgical pituitary specimen concerns as to whether the tissue submitted for analysis is normal pituitary or a pituitary tumour. For this, the most helpful histochemical stain after hematoxylin-eosin (HE) is the reticulin technique, which helps to differentiate the preserved acinar pattern of normal adenohypophysis from the disruption of the reticulin network seen in pituitary tumour. The second decision to be taken will be if the lesion is a tumour or not, because the pituitary may be affected by different lesions, some with similar characteristics of a pituitary tumour. Pathology provides a significant contribution to the Pituitary MDT by offering detailed information about the histological and morphological characteristics of pituitary lesions (pleomorphism, mitoses, inflammatory changes, stroma, haemorrhage, vascular features or proliferative index). This information can help to better understand the nature and degree of aggressiveness of the lesions, which can influence decision-making and treatment plans. Moreover, pathology provide insights about specific molecular markers that may be relevant for diagnosis and selection of targeted therapies, in a standard report indicating the final diagnosis."
**Neuro-ophthalmologist**	"Neuro-ophthalmologists can play an important role in the early diagnosis of pituitary tumours, as blurred vision or visual field loss may be the first presentation. Diplopia may also occur due to compression of the third, fourth or sixth cranial nerves in the cavernous sinus. These patients may be otherwise healthy, with no headaches or neurologic symptoms. Neuro-ophthalmologist input is also critical for the treatment decisions. Neuro-ophthalmologic evaluation is essential to decide the need and urgency for pituitary surgery, and in some cases, emergency surgery may be required if the visual loss is rapidly worsening and/or severe, or if the tumour is pressing the optic chiasm. Pre-operative assessment helps predicting the visual prognosis. Normal pre-operative optical coherence tomography is associated with a higher visual recovery rate, whereas pre-operative thinning of the retinal nerve fiber layer or ganglion cell layer correlates with a poorer visual prognosis. Neuro-ophthalmology follow-up should be continued for several years, as the first (and sometimes only) sign of tumour recurrence may be new-onset or worsening of visual field defects."
**Otorhinolaryngologist**	"Otorhinolaryngologists play a secondary role on a Pituitary MDT. On an otorhinolaryngology consultation a pituitary patient may complain of headaches suggesting sinusitis, or hyposmia, both being possible presentation symptoms of pituitary disease. However, most of the patients referred to an MDT from

	an otorhinolaryngologist will be for an incidental finding on imaging, requested frequently for sinonasal disease. From a treatment perspective, this specialty participates on the surgical evaluation as most patients will be operated through an endonasal approach. The planning of the surgical route entails the evaluation of the least traumatic pathway, the way to obtain the clearest view avoiding damage to critical structures, and also to plan the reconstruction especially when flaps are needed to close an eventual fistula. During surgery, the endonasal part is often performed by an otorhinolaryngologist, while otorhinolaryngologists are also relevant at the end reconstruction phase to deploy the flaps, review the hemostasis, reposition the nasal structures to prevent nasal obstruction or hyposmia after surgery. Some cases benefit from minor procedures to avoid sinusitis or lacrimal duct obstruction. After surgery, at the outpatient clinic, patients are observed regularly and some procedures may be performed to speed up the healing processes and allow a faster return to normal, such as removal of obstructive crusts or resolution of synechiae. Also, early post-operative observation may allow the prompt diagnosis of complications such as cerebrospinal fluid leak, sinusitis, reconstruction flap necrosis, or mucocele formation."

Supplementary Table S2: The perspectives of different specialists involved in our Pituitary MDT regarding the question "What are the major contributions that other specialties can provide to your clinical practice when managing a pituitary tumour patient?"

**Medical specialist**	**What are the major contributions that other specialties can provide to your clinical practice when managing a pituitary tumour patient?**
**Neurosurgeon**	"Neurosurgeons dedicated to pituitary diseases may experience new insights to their own surgical skills when enrolled in a multidisciplinary discussion to promote a better understanding of many nuances from the other members of the team. Neurosurgeons also introduce their colleagues into meaningful aspects of pituitary surgery. Together we can decide on the best therapeutical approach for each case. Confidence between team members is key to achieve the best outcomes for the patients, and all the colleagues are important in the different disease stages; for instance, endocrinologists are important in the pre- and post-operative assessment of the pituitary function and if necessary in replacing missing hormones or control excessive hormone levels in functioning tumours; neuroradiologists are key in helping to plan the surgery and in the long term follow-up in interpreting serial imaging studies; neuropathologists are key for the final diagnosis after the operation; etc."
**Endocrinologist**	"Endocrinologists rely on the expertise of several medical specialties: i) neurosurgery plays a crucial role in surgical interventions, ensuring optimal tumour removal while preserving neurological and pituitary function; ii) neuroradiology offers expertise in interpreting imaging studies, which are essential for diagnosing, monitoring and deciding the best treatment strategies; iii) neuro-ophthalmology is indispensable in diagnosing and managing visual disturbances caused by tumours compressing the optic pathways; iv) neuropathology enables the precise diagnosis and characterization of the pituitary tumour from a histological point-of-view, providing key insights in its hormone-secreting subtype and its potential

	aggressiveness, which may be relevant in guiding therapeutic approaches. In addition to these specialties, other professionals can play a vital role in the care of pituitary tumour patients, including endocrine nurses. By working together, the MDT can offer comprehensive and patient-centered care, addressing both the tumour itself and the patient's overall health and well-being.”
**Neuroradiologist**	“When managing a pituitary tumour patient in a Pituitary MDT there are important information from the team that can optimize the neuroradiologist work. Effective communication is crucial as well as keeping up-to-date records in the MDT files. If a patient presents for a scan, neuroradiologists check the relevant history that may guide the evaluation, including symptoms, ophthalmology observations, previous treatments, especially those related to pituitary disorders. Referring physicians should specify the questions or goals for requesting the study and the intended course of action following the result. If available, previous scans should be provided for comparison. It is important to refer for claustrophobia, metal implants that may not be compatible with MRI, renal dysfunction, allergies or previous adverse reactions to intravenous contrast. An important contribution from neuropathology and neurosurgery is to know the final diagnosis and tumour characteristics during surgery. The same is true concerning type of surgery and post-surgery material for sellar/sphenoidal sinus flap or patch. All these data will help the neuroradiologist to accurately read the scan. The feedback after treatment improves the neuroradiologist knowledge and experience and the clinical goals of the Pituitary MDT.”
**Neuropathologist**	“The pituitary may be affected by a wide range of conditions having similar clinical characteristics. Other medical specialties can provide valuable contributions to the histopathologic diagnosis when managing a pituitary tumour patient. Endocrinologists can offer expertise in hormonal evaluation and management, helping to assess the functional status of the tumour and its impact on the endocrine system. Neuroradiologists contribute with interpreting imaging studies to assess tumour size, location, and potential invasion into surrounding structures. Neurosurgeons play a crucial role in surgical interventions, providing insights into the feasibility and potential risks of surgical resection. Additionally, oncology specialists may provide guidance on the use of targeted therapies or radiation therapy in cases of aggressive or metastatic tumours. Collaboration between these specialties allows pathologists to integrate clinical and radiological information with histopathological findings, leading to a more accurate diagnosis, prognosis, and treatment plan for the patient.”
**Neuro-ophthalmologist**	“After resection of a pituitary tumour, there is often a marked recovery of visual function, which tends to occur rapidly after the surgery (patients sometimes report improvement in vision immediately after waking up from anaesthesia), and further improvements may continue over the next months, provided that optic atrophy is not advanced. Thus, the role of neurosurgeon and effective surgery is key for visual outcomes of patients with large tumours compressing the optic chiasma. Other tumours of the pituitary region are more difficult to treat, and visual prognosis is less certain. Referral to a neuro-ophthalmologist after diagnosis and throughout treatment is essential to evaluate and monitor pituitary tumour repercussions on visual function, and the information provided by the neuroradiologist regarding the relationship of the pituitary tumour with the optic apparatus in the scans is crucial for the best interpretation of neuro-ophthalmologic evaluations.”
